# Exploring risky driving behavior and its underlying factors: a qualitative study in Iran

**DOI:** 10.5249/jivr.v17i1.1953

**Published:** 2025-01

**Authors:** Reza Fereidooni, Ahmad Kalateh Sadati, Seyyed Hamidreza Ayatizadeh, Saeed Shahabi, Yaser Sarikhani, Seyed Taghi Heydari, Kamran Bagheri Lankarani

**Affiliations:** ^*a*^ Institute of Health, Health Policy Research Center, Shiraz University of Medical Sciences, Shiraz, Iran.; ^*b*^ Department of Social Sciences, Yazd University, Yazd, Iran.; ^*c*^ Trauma Research Center, Rajaee (Emtiaz) Trauma Hospital Shiraz University of Medical Sciences, Shiraz, Iran.; ^*d*^ Research Center for Social Determinants of Health, Jahrom University of Medical Sciences, Jahrom, Iran.

**Keywords:** Aggressive driving, Distracted driving, Traffic accidents, Risky Driving Behavior, Iran

## Abstract

**Background:**

Risky driving behavior (RDB), a major contributor to road traffic injuries, is a complex issue with multiple dimensions. This study aimed to explore the experiences and perspectives of drivers who engaged in risky driving behaviors in Shiraz, Iran.

**Methods:**

In 2023, we conducted a qualitative study in Shiraz, Iran, with 35 drivers whose licenses were revoked for traffic violations. Through semi-structured interviews, we examined specific instances of high-risk behaviors, prompting drivers to recall the factors that led to their actions. Additionally, the questions explored the impact of various determinants of risky driving, drawing on participants' personal experiences. The data were analyzed using the conventional content analysis method.

**Results:**

The findings revealed that various factors, ranging from individual to structural, contribute to the formation of RDB. We identified four themes: job conditions, personal traits, socio-cultural factors, and infrastructural factors. Economic pressures and employer-imposed time constraints contributed to risky driving, while impulsivity and thrill-seeking tendencies played a role at the individual level. Social norms, peer influence, and perceptions of arbitrary law enforcement were the sociocultural risk factors, and poor quality roads and inadequate traffic monitoring were infrastructural factors that contributed to reckless driving.

**Conclusions:**

This research underscores the interplay of economic challenges, job-related pressures, social dynamics, and personal characteristics in shaping RDB. Additionally, it sheds light on previously underexplored aspects which have implications for policy, traffic authorities, and driver training programs aimed at enhancing road safety in Iran.

## Introduction

Road traffic accidents (RTAs) are a major cause of morbidity and mortality worldwide. They are especially prominent in low- and middle-income countries (LMICs).^1^ Road traffic fatalities in LMICs account for 90% of global road deaths.^2^ Rising incomes in these countries have driven a rapid increase in motorization, outpacing road safety management and regulations. In 2015, the death rate from road traffic injuries in the LMICs was 34 per 100,000.^2^ In Iran, road traffic injuries have had a generally increasing trend in recent decades.^3^ However, RTA-related mortality in Iran peaked in 2005, began to decline, and nearly plateaued in recent years, with 20.7 deaths per 100,000 in 2019 and a decline in 2020, likely due to COVID-19 travel restrictions.^3^

Most studies indicate humans as the key cause of RTAs.^2,4^ A wide range of factors, including individual characteristics like age, gender, personality traits, and cognitive abilities, as well as external factors like the environment, traffic conditions, and vehicle characteristics, can influence driving behavior.^5^ The determinants of human factors leading to risky driving behavior (RDB) are numerous but have been identified at a general level. In a review, Jafarpour and Rahimi Movaghar found numerous and complex factors affecting risky driving, including gender, age, driving experience, physical and mental health, personality traits, moods, emotions, socio-economic background, income level, and the impact of law enforcement on societal behavior regarding road rules.^6^ Researchers have determined the impact of environmental factors such as roads and weather conditions on crashes;^7,8^ however, their relationship with driver behavior remains unclear. Frequent exposure to traffic congestion is a recognized factor that can lead to frustration and aggressive behavior.^9^

A wide range of factors, including personal experiences, cognitive processes, beliefs, social norms, situational variables, and personality traits, influence human behavior.^10^ Therefore, we cannot reduce RDB, a complex human behavior, to one or a few causes or explanations. Conducting interviews with high-risk drivers is a valuable approach to gaining insights into their driving behavior, as it enables researchers to enhance their comprehension of the drivers' perceptions, attitudes, experiences, and motivations that underpin their actions.^11^

A study from Australia determined that speeding, habitual or unintentional, and distraction, particularly with mobile phones, were the most commonly reported RDB emerging from interviews.^12^ Researchers in Ethiopia conducted a study in 2021 to explore drivers' perceptions of RDB in public transport vehicles. They identified themes of “Safety Rule and Enforcement Issues,” “Drivers' Training Curriculum and Application Gaps,” “Technical and Financial Problems,” and “Passenger and Vehicle Owners' Influence”.^13^ A multi-method study from the Czech Republic used interviews with drivers involved in accidents to determine the individual factors leading to the accident. However, they did not further investigate the underlying causes.^4^ A qualitative study among 25 US college undergraduate students revealed that social circles and norms significantly impacted their driving behavior. Barriers and the perceived impoliteness of intervening, made texting and driving a prevalent issue.^14^ The study by Day et al.^15^ determined what leads to a reduction in crash risk among novice drivers; improvements in car control and situation awareness, a reduction in thrill-seeking behavior, and early concerns about social status influencing driving speed and risk-taking. A qualitative study in Tehran with 42 taxi drivers explored their views on risky driving behavior and ideas for a social marketing program to reduce it, emphasizing the need for persuasive messaging to improve driver attention and behavior.^16^

Iran presents a unique traffic landscape due to its high vehicle density, with most cars being locally manufactured and often of questionable quality. Additionally, its vast geography results in an extensive and diverse road network, further influencing driving behavior and road safety challenges. RDB is a major threat to road safety, and while extensive research exists on this subject, our qualitative understanding of its root causes and motivations remains limited. Semi-structured interviews are also suitable for this type of research because they offer the drivers the opportunity to discuss the multiple factors influencing their behavior. This approach enables the researcher to explore not only the drivers' motivations but also the broader socio-cultural, psychological, and situational elements that contribute to risky driving leading to a comprehensive understanding of the problem, which would be difficult to capture through surveys or other quantitative methods. This qualitative study aims to explore the backgrounds and underpinnings of RDB by interviewing high-risk drivers and going beyond a superficial understanding of risky driving behavior by engaging with high-risk drivers. 

## Methods 

**Setting and participants**

This study took place in Fars Province, located in southern Iran. The focus was on high-risk drivers whose licenses were revoked by the Fars traffic police in 2021, usually as a penalty for repeated dangerous violations.

 Participants were selected from a list provided by the Fars Province Traffic Police. Exclusion criteria included drivers who did not answer their phones after three call attempts, those who could not speak Persian, and those who did not provide consent to participate. 

We continued sampling until we achieved data saturation and refined the themes. Forty-eight drivers were contacted. A total of 35 participants, including 19 light vehicle drivers and 16 heavy vehicle drivers, agreed to participate in the study. Light vehicles included passenger cars (sedans or SUVs), vans, and pickup trucks, while heavy vehicles encompassed trucks (lorries), buses, and trailer trucks. If participants owned both types of vehicles, the type they drove most frequently was considered their primary vehicle.

**Data collection**

We conducted phone calls to several high-risk drivers whose driving licenses had been revoked by the traffic police. After discussing the research objectives, we included those who expressed a willingness to participate in the study. We established a location for the interviews in coordination with the participants, and conducted semi-structured interviews. Data collection began on June 12, 2022. The semi-structured interviews utilized an interview guide that included open-ended questions and suggested probes. Our interview guide is presented inTable 1. The questions were designed to elicit specific instances and personal experiences related to risky driving, encouraging drivers to share their motivations. After these initial questions, we followed up with more targeted inquiries to explore a range of factors influencing risky driving, including topics like job-related influences, economic standing, the role of law enforcement, and the impact of other drivers.

**Table 1:** Interview guide.

Question	Probing question
1. Remember the times you were fined for speeding, was there any particular reason for speeding?	What led to that reason for speeding?
2. Remember the times you were fined for unauthorized overtaking, was there any particular reason for overtaking?	What led to that reason for overtaking?
3. How does your job affect your driving?	Your experience? Does job condition, working hours and time of day affect your driving? How?
4. How does your economic standing affect your driving?	How? Your experience?
5. How do the police and laws affect your driving?	Your experience with law enforcement? What can the police do to improve your driving?
6. How does other people’s driving affect your driving?	Your experience?
7. What has lead you to drive under the influence of alcohol or substances?	Why do you drive under the influence despite knowing the dangers?
8. How do poor road and weather conditions affect your driving?	How? Your experience?

Incorporating both exploratory and descriptive elements allowed us to gain a comprehensive understanding of the factors behind risky driving behavior.

**Data analysis**

For the synthesis of data, the conventional content analysis method was applied, utilizing the six-step methodology suggested by Graneheim and Lundman.^17^ This methodology comprised familiarizing oneself with the data, extracting initial codes, developing themes, reviewing those themes, defining them, and reporting the results.^17^ Two researchers independently examined the transcripts, reconstructing the texts into meaning units before classifying them into codes and subthemes, following the content analysis technique. The research team then discussed the subthemes and abstracted them to assign the parent themes. In order to reduce the possibility of bias, data collecting and analysis techniques included critical reflexivity.^18^ Each interview began with the interviewer describing the objective of the project and profession and the body of work of the first author. In addition, the writers participating in the analytic process had diverse academic backgrounds, which might lessen bias. 

In qualitative research, it is crucial to align the chosen level of abstraction for the extracted themes with the research objectives and the complexities of the findings. In the context of a study involving drivers, it is crucial to consider that the expected results may not align with high-level abstractions. Consequently, although our themes were relatively broad, the subthemes were notably concrete.

**Trustworthiness measures**

To ensure the validity and rigor of the study results, we incorporated the four criteria proposed by Guba and Lincoln: credibility, dependability, confirmability, and transferability.^19,20^ To enhance the credibility of our results, we committed to an extensive engagement with the data and facilitated opportunities for external validation throughout all phases of the study by involving two colleagues external to the project in a peer debriefing process. Additionally, we employed a peer-check technique as a method of triangulation. Two researchers conducted independent analyses of the interviews and subsequently compared their findings to identify both similarities and discrepancies. A coordinated meeting was conducted with all researchers to review the sub-themes and key themes. We aimed to diligently document all stages of the study to enhance its dependability. In this regard, the protocol and methodology of the study were scrutinized by two external reviewers. We aimed to enhance the confirmability of the findings by inviting two external qualitative researchers to confirm the accuracy of the study protocol and the data synthesis. To ensure transferability, we provided a thorough description of the research procedure.

**Ethical considerations**

We invited all participants through an introductory phone call. The in-person interviews required participants to read and sign a written consent form that detailed the project's objectives and aims in Persian. We explained the purpose of the telephone interviews at the beginning, asked participants if they had free time to answer the questions, and requested verbal consent. All participants were free to withdraw from the study at any stage. We assigned numbers to participants during transcription and analysis to maintain their anonymity. Approval for the study protocol was obtained from the Ethics Committee of Shiraz University of Medical Sciences (code: IR.SUMS.REC.1401.043).

## Results

Data analysis revealed that participants demonstrated a thorough understanding of the risks associated with high-speed and reckless driving. They also clearly identified the underlying reasons for engaging in such behaviors. Based on their insights and experiences, job-related conditions emerged as a significant contributing factor. High-speed driving and reckless behavior were found to be influenced by a combination of factors, including personal traits, social and cultural dynamics, and insufficient or poorly developed infrastructure. Except for one participant, all drivers stated that they did not consume alcohol. The individual who did consume alcohol mentioned that he actively manages his time to minimize the risk of accidents.

The data analysis identified four main themes: job conditions, personal traits, socio-cultural factors, and infrastructural factors (Table 2).

**Table 2:** Themes, subthemes and concepts explored related to risky driving.

Themes	Sub-themes	Initial concepts
**Job conditions**	**Economic challenges**	The cost of living, expensive vehicle spare parts, low income, working extra hours for more pay, increasing fuel prices, low pay
	**Time pressure**	Time constraints, driving without breaks, urgent delivery of export shipments, heavy workload, Employer/client requires prompt delivery of cargo, rushing to deliver goods
	**Poor working condition**	Unsustainable for older drivers, lack of sleep and rest, excessive working hours, solitary driving, delayed retirement, monotonous work routine, fatigue, boredom, and insomnia.
**Personal traits **	**Emotional regulation**	Feelings of nervousness, irritation, frequent arguments, quick temper, stubbornness while driving, selfish behavior, lack of self-control, and absence of calmness.
	**An occupied mind**	Losing focus, unintentionally exceeding the speed limit, distractions, absent-minded overtaking, busy thoughts
	**Risk personality and perception**	Thrill of speeding, sensation seeking, excitement from zigzagging, learning from others' mistakes, new drivers' risk awareness, experience reducing driving errors, lack of awareness of road hazards, habitual speeding
**Socio-cultural factors **	**Cultural lag **	The role of family in shaping culture, cultural values versus financial gain, high-speed subculture, cellphone usage while driving, slow driving in the fast lane, habitual speeding, disregard for traffic signs,
	**Arbitrary law enforcement**	Unjust fines, inconsistent and ineffective law enforcement, inconsistent enforcement of rules, the police are stricter with heavy vehicles, negative attitude from the police, lack of positive reinforcement, unlucky to get caught, everybody does it
	**Peers**	Lack of good role models, the influence of driving companions, non-compliance of other drivers, social learning, the presence of reckless drivers like smugglers on rural roads.
	**Poor driving literacy **	Public not well-informed on rules, lack of adequate education, police officers are not adequately trained or well-informed on regulations, lack of informing the public through media, drivers not well-trained
**Infrastructural and environmental factors**	**Environment**	Speeding on unattractive and deserted roads, extreme temperatures impair driving abilities, hot weather leads to impatience
	**Poor quality roads**	No highways, low-quality asphalt/roads, slow traffic due to congestion, obsolete infrastructure
	**Lack of control agents **	Absence of police, lack of police presence, the deterrent role of police presence, fines serve as an incentive to follow rules
	**Substandard Vehicle **	The low quality of Iranian cars, technical defects in vehicles, faulty air conditions, faulty speedometers

**Job Conditions**

The first theme refers to poor job conditions, which encompass general aspects such as the type of work, low income, vehicle maintenance costs, and strenuous work. All these conditions lead the driver to drive at high speed. 

The participants highlighted the challenging economic conditions and the imbalance between income and living expenses as significant issues. Many participants agreed that financial status influences various behaviors. The cost of living, car repairs, and income and expense disparities have compelled individuals to drive for extended hours, leading to fatigue, drowsiness, and an increased risk of accidents. 

Many participants expressed their concern regarding excessive workload. This theme was especially prominent for heavy vehicle drivers who usually drove on intercity roads, which entails its own set of challenges. They also attributed their excessive workload to rising living costs and a struggling economy, which compelled them to undertake additional deliveries or trips. In addition, they faced time pressure to meet delivery deadlines, as their employers demanded the transportation of cargo within a less-than-optimal time. A significant number of our heavy vehicle driver participants indicated that they do not actually own the vehicle. However, they are obligated by the vehicle owner to fulfill excessive transport, which allows both the owner and the driver to meet an income target. They attributed some of their violations to driving at high speeds or while fatigued, citing the previously mentioned reasons.

Heavy vehicle drivers also expressed concerns about the physical toll of their work, emphasizing issues such as back and neck pain caused by the demanding nature of their jobs. They emphasized that such conditions were unsustainable over an extended period and contributed to lapses in concentration and diminished alertness while driving.

*Participant no. 3: “Sometimes, I drive for 24 hours straight because the expenses have gotten so high that my 8-hour job doesn't cover the cost of living. We're forced to work day and night for my wife and child to make ends meet”.*

*Participant no. 8: “My low income compels me to work an extra 5 to 6 hours a day, leading to fatigue, nervous problems, and accidents."*

*Participant no. 29: “The job is heavy. It is not sustainable for thirty years. I have back and neck pain. The career span of a functioning driver should not exceed 20 years”.*

*Participant no. 11: “At one point, for example, the employer calls and instructs you to leave at a certain time. Our job is to export; we have to get the cargo to the ship. Because of this. They call and insist that we arrive on time”.*

**Personal traits**

This theme highlights the conditions and personal characteristics that lead to RDB. 

The participants emphasized that anger and frustration can affect driving performance. An individual with a tendency to become frustrated easily may react aggressively when faced with a driving problem, resorting to speeding, overtaking, and zigzagging, which can lead to accidents. Such a person may also misinterpret the behavior of other drivers and react with stubbornness and anger. These characteristics not only result in dangerous driving but also create hazardous conditions on the road by interacting negatively with other drivers.

Another factor is a preoccupied mind. Economic problems, work pressure, and job issues can distract drivers to the point where they are unaware of their speed. Some participants noted that when their minds are occupied, they tend to 'zone out' while driving, leading to unintentional speeding. Boredom and impatience are also significant contributors. In urban areas, these feelings cause drivers to swerve through traffic openings. On rural roads, being stuck behind slow-moving vehicles often leads to overtaking in prohibited areas.

 Risk personality and risk perception are another subtheme. A "risk personality" refers to an individual's tendency to engage in behaviors that involve taking risks. Excitement-seeking and habitual speeding are additional factors related to risk personality, indicating a personal inclination towards thrill and a tendency to drive at high speeds. Individuals with a sensation-seeking personality enjoy speeding, weaving through traffic, and zigzagging, often disregarding the rights of others and traffic rules. This behavior can lead to accidents or cause vehicles to overturn. Traits such as excitement-seeking, having less driving experience, and being a novice driver increase the likelihood of risky driving. Perception of risks, in this context, refers to a lack of awareness of road hazards or a limited understanding of these dangers, which can lead to unlawful behavior. This also involves underestimating or disregarding risks, as some drivers believe they are above the law due to their driving skills or a belief that they "know better." New drivers, particularly those who have been licensed for several months to a year, are more prone to risky driving behaviors compared to both recently licensed and experienced drivers, probably due to risk unawareness stemming from inexperience.

*Participant no. 5: “I have become accustomed to driving at high speeds. Because I am used to driving fast, I find it challenging to drive slowly even when I want to”.*

*Participant no. 1: “I did not notice the high speed. Maybe it was due to distraction or mental preoccupation”.*

*Participant no. 17: “I myself now comply more. As our work experience has increased, we have become more compliant. Even hearing our colleagues driving experiences had made us better drivers”.*

**Socio-cultural factors**

Social and cultural factors are said to reinforce RDB. Initially, the drivers emphasize the impact of cultural lags. All participants emphasized that the society is yet to catch up with the necessary changes in attitudes, highlighting that some risky driving behaviors are ingrained in cultural norms that are typically learned from childhood. In the context of poor driving literacy, in some cases, drivers are simply unaware of certain traffic laws due to insufficient education or training. In other instances, drivers may be aware of the rules but choose to ignore them because it's more convenient or they feel it won't result in consequences.

Peer influence from driving companions and other drivers can encourage risky behavior, such as speeding or ignoring traffic laws. Drivers may mimic the actions of those around them, especially if reckless behavior is normalized within their social circle or in the traffic environment. Even the passengers in the car sometimes exert influence on the driver through their actions, remarks, or requests, prompting the driver to accelerate despite their desire for caution. 

Some participants mentioned the belief of "everyone does it, so I will too," or the idea that engaging in risky behavior is necessary to defend one's rights where others disregard the rules. This highlights the strong influence of peers on driving, as it pressures individuals to adopt unsafe practices, reinforcing a cycle of risky behavior within social groups.

Another point of emphasis for the drivers is the presence of unfair and arbitrary laws enforced by the police. The participants mentioned that there is a perception of inconsistent enforcement of traffic laws, where certain individuals or vehicle types are more likely to be fined for offenses while others seem to get away with similar violations. They also complained that many roads lack constant monitoring, so when police decided to set up a speed control, they were “unlucky” to get caught. In fact, 11 of the participants deemed their fines to be “unjust.” Apart from the absence of continuous surveillance, individuals experienced a sense of unfairness for various other reasons. These included a prevailing perception that everyone else was engaging in similar behavior, a belief that the offense in question was too trivial to warrant a fine, even if it still technically constituted an offense, and a view that the existing laws lacked logical reasoning. Furthermore, a number of the drivers have expressed they have “talked their way out of a ticket” or negotiated a lower amount of fine.

*Participant no. 30: “I know intersections where everyone violate the red light and encroach the opposite lane, but since there’s no police to control it, people continue to do it.”*

*Participant no. 13: “If you commit any offense with an Iranian car, they will let it pass, but if it is a foreign car or a heavy vehicle, you must be fined. There should be no difference; the law must be enforced in society, and everyone should comply.”*

*Participant no. 18: “When I see many drivers illegally overtake and violate my rights, I have to overtake myself.”*

*Participant no. 16: “Everyone violates abundantly and sometimes one gets unlucky and get fined for a violation.”*

**Infrastructural and environmental factors**

The final theme pertains to external factors of environment and infrastructure including traffic control, vehicle quality, roads and weather condition. 

One primary concern was the poor quality of the roads. The roads have dangerous curves, broken asphalt, and potholes, which can lead to accidents, veering off the road, or vehicle overturning. Therefore, poor road quality was mentioned as affecting driving behavior. Drivers reported that they try to make evasive maneuvers to avoid potholes or road cracks, particularly when they encounter them unexpectedly. This involved sudden braking, abrupt lane changes, or risky maneuvers such as swerving into oncoming traffic. Lack of clear signage was another concern; some signs are either distorted due to their age, or the laws have changed, but the signs have not been updated. Traffic congestion, caused by poorly designed roads, was commonly described as frustrating due to time loss and the need for constant adjustments between braking and accelerating. Lane changes during traffic jams were particularly irritating, especially under time pressure or when returning home after work. Consequently, many drivers reported increasing speed and overtaking to compensate for the delays.

Almost all drivers acknowledged that adverse weather conditions like rain, snow, and fog contribute to accidents, prompting them to drive more cautiously and avoid overtaking or speeding. But the interesting finding is that the hot climate in southern Iran leads to fatigue and impatience, reducing focus. Drivers also mentioned avoiding the use of air conditioner to prevent car depreciation, as many vehicles are of low quality. 

Many of the cars lack proper safety features and some have faulty speedometers, leading drivers to be unaware of their speed. Several participants expressed dissatisfaction with the performance, reliability, comfort, and safety of their vehicles.

*Participant no. 28: “One gets stuck behind slow heavy vehicles, and you can’t overtake because there are so many cars on the opposite lane or overtaking is prohibited in many places. It would be much better if they built a roads with more lanes”.*

*Participant no. 25: “The condition of the roads to the south is very bumpy. To avoid falling into a pothole, the driver must signal properly; failure to do so may result in an accident”.*

## Discussion

Discovering the underlying factors and backgrounds that lead to RDB is crucial for enhancing traffic safety. Quantitative studies have determined some traditional factors, but we also need to consider the subjective underpinnings of RDB. We discovered four themes: job conditions, personal traits, socio-cultural factors, and infrastructural factors. Our novel findings include the impact of time pressure from employers, underdeveloped roads, and surface defects that hinder overtaking and vehicle control. Our findings indicated that arbitrary law enforcement led drivers to perceive risky behaviors not as serious due to inconsistent consequences. Additionally, drivers often experience mental distractions, primarily related to economic stress, which leads to absent-mindedness while driving. Hot weather was also found to contribute to restlessness, further affecting driving behavior.

Job conditions including low income, overworking, time pressure, and poor working conditions shed light on the complex interplay between economic factors, job demands, and driving behavior. Previous research has shown mixed associations between economic factors and RDB.^21-23^

Economic pressures like low income and financial strain often push drivers to maximize their earnings by taking on more work, leading to behaviors such as speeding, ignoring traffic regulations, or driving fatigued to meet deadlines. Tight deadlines and heavy traffic contribute to time pressure, which previous research links to higher speeds and increased physiological responses such as elevated heart rate and reduced blinking.^24^ It is also known that time constraints as a contributing factor to risk-taking in driving.^25,26^ A study in Nigeria^13^ also reported that time pressure from employers, contract providers, or vehicle owners is a significant issue in LMICs. Previous research has also indicated that a sense of time pressure is more prevalent in professions that require chronic driving.^25^

We also found that poor working conditions, especially among heavy vehicle drivers, affect both physical and mental well-being, contributing to RDB. Mental strain, as explained by the theory of situation awareness^27^ reduces attention and may cause drivers to miss crucial cues, such as road signs or nearby vehicles, leading to misinterpretation of potential hazards. As shown in previous research, physical discomfort, such as back and neck pain, can lead to impaired cognitive function while driving, as shown in drivers with chronic back pain.^28,29^

In conclusion, economic challenges significantly affect driving behavior in several ways: first, insufficient income leads to fatigue from longer work hours; second, economic concerns create a “preoccupied mind”; third, time pressure exacerbates risky driving; and fourth, poor working conditions compound the issue. Understanding these factors is essential for developing effective interventions, as focusing only on education or law enforcement may not be enough, especially in low- and middle-income settings. 

There are numerous studies on the impact of mental state, mental disorders, and personality traits on driving behavior. Stressors are known to lead to worse driving habits. RDB is known to positively correlate with personality traits such as anger, hostility, sensation seeking, impulsiveness, and boredom.^30^ Consequently, two of the drivers expressed that they find speeding enjoyable, a trait associated with sensation-seeking or excitement-seeking. In our study, participants reported that boredom motivated them to overtake slower drivers. Heslop et al.,^31^ suggest that while drivers may link boredom to excitement seeking, other factors, such as a lack of stimuli, also contribute to feelings of boredom. Drivers also shared instances where emotional turmoil or distractions compromised their ability to focus on the road. Drivers attributed their lack of focus to economic problems and "life hassles." We used “occupied mind” instead of distraction, since the term distraction may refer to behaviors such as cellphone use or eating while driving. In line with our findings, a study on taxi drivers in San Francisco showed hassles and job-related stress contribute to drivers’ depression symptoms.^53^

Regarding the subtheme of risk insight, some studies have confirmed the association between risk perception and engagement in RDB such as drunk driving.^32,33^ Researchers believe that many factors, including age, gender, driving experience, social norms, personality traits, and personal beliefs, influence risk perception.^33-36^ Similarly, the driving experience was a subtheme among our participants. Additionally, perceptions of risk and skill, specifically the underestimation of risks and the overestimation of driving skills, can influence RDB. While low skill can lead to aberrant driving, some studies have indicated that overestimation of driving skill can lead to unsafe driving.^37^ One study demonstrated a correlation between overconfidence in one's perceptual-motor skills and both aggressive and ordinary violations.^38^ Such drivers may be less likely to recognize their limitations, perceive potential dangers, or acknowledge the consequences of their actions. They are also prone to multitasking, like using mobile phones while driving.

Personality traits such as extraversion and narcissism may play a part in the behavior of these individuals as they try to assert dominance on the road and showcase their perceived driving prowess.^39^ Social norms^6,14^ or social learning^40,41^ can explain how other drivers influence a driver's behavior. Drivers may feel compelled to conform to norms when they observe others widely practicing or accepting certain behaviors. Social learning theory suggests that individuals learn behaviors by observing and imitating others. When drivers observe other drivers engaging in specific behaviors, they may learn from those observations and incorporate them into their own driving behavior.

Socio-cultural factors are associated with driving norms and values among drivers, which can lead to RDB. Although the mere presence of passengers may not affect the driver’s behavior significantly,^42^ it may happen if it leads to distractions, increased stress, or influence the driver's decision-making.^43^ For example, it was shown that a driving companion who is accepting of drunk driving can influence the driver's behavior, leading to higher risky driving behavior.^44^ Also, a driver’s decision-making may become disturbed if their companions are suggesting that they show a certain driving behavior such as “going faster”.^43^ This can lead to a higher risk of accidents, as the driver may not be fully focused on the road ahead. Similarly, our participants said that the presence of certain passengers lead to an “escalation” of excitement and risk-taking behaviors. The influence of other drivers on the behavior of the driver can be explained by social norm^6,45^ or social learning.^40,46^ When drivers observe certain behaviors being widely practiced or accepted by others, they may feel compelled to conform to those norms. Social learning theory suggests that individuals learn behaviors by observing and imitating others. When drivers observe other drivers engaging in specific behaviors, they may learn from those observations and incorporate them into their own driving behavior.

Arbitrary law enforcement can be defined as inconsistent or discriminatory enforcement and should be differentiated from selective enforcement programs, which refer to special attention to one specific behavior in terms of education, public awareness, and enforcement.^47^ Arbitrary enforcement practices and leniency can undermine the effectiveness of law enforcement efforts by eroding trust in the system and reducing the perceived risks associated with engaging in RDB.^48^ Our findings regarding the inconsistency and arbitrary enforcement of safety regulations align with the findings of the qualitative study by Mazengia et al.^13^ This theme in Iran may arise from the practice of negotiating traffic tickets and the inconsistent presence of law enforcement, leading to the belief that certain behaviors are not always punished.

On the theme of infrastructure factors, the subthemes of roads, law enforcement, and environment come into play. Research has shown that potholes and rough road conditions influence driving behavior. When drivers come across these road hazards, they often need to slow down to protect their vehicles and maintain control.^49^ Being aware of these dangers can make drivers overly cautious or prompt them to find alternative routes. Potholes can create sudden jolts that pose risks of losing control or swerving into other lanes. Additionally, poor road conditions result in a bumpy ride, making it challenging to maintain a steady speed.^50^ Poor road conditions disrupt the smooth flow of traffic or can cause damage to vehicles, including tire punctures, wheel misalignment, or suspension damage. Frequent encounters with such conditions can increase driver stress and fatigue. Navigating around potholes and cracks demands heightened attention and vigilance, which can be mentally and physically draining. Traffic congestion is often exacerbated by inadequate road infrastructure. The finding of frustration in traffic congestion closely mirrors David Shinar’s frustration-aggression hypothesis.^9^

One aspect of infrastructure is law enforcement, particularly how widespread and effective its implementation is, and how well law enforcement agencies are able to function in ensuring road safety. Visible police presence encourages drivers to adhere to traffic laws, as it instills a fear of detection and penalties for their actions. This finding aligns with existing research on the role of law enforcement in promoting road safety and safe driving behavior.^51,52^ The lack of presence of officers in areas with frequent traffic violations may indicate a gap in enforcement strategies or resource allocation. More attention and resources should be allocated to monitoring high-risk areas to effectively reduce RDB. Improving the visibility and frequency of police patrols at these locations may help mitigate violations and enhance road safety. Enhancing road infrastructure can alleviate frustration by reducing traffic congestion, adding more legal overtaking lanes where drivers don't need to encroach on the opposite lane, and improving road signage for clearer navigation. Moreover, faster travel times due to smoother roads and better traffic flow would help reduce stress, enabling drivers to maintain focus and make safer decisions on the road. A novel environmental factor in our study is that hot weather, often overlooked in previous research, can lead to restlessness and exhaustion, contributing to risky driving. This is a subject worth considering in designing vehicles for countries with such climates.

The findings of this study can also reflect the theory of planned behavior, which suggests that three factors attitude, subjective norms, and perceived behavioral control shape an individual's intention to engage in a specific behavior.^53^ Subjective norms and the idea that you have control over your behavior could be linked to the study's themes of peer pressure^54^ and self-perception of driving abilities.^55^ Attitude, on the other hand, refers to beliefs about the behavior and the associated outcomes, which can reflect the risk perception subtheme.^33,56^ In the context of this theory, studies suggested driver education and campaigning to approach RDB.^57^

Many of the issues identified, particularly those related to job and economic conditions, can be addressed through broader policies targeting the economy. Improving transportation infrastructure and enforcing industry standards in vehicle production are also vital. Although large-scale improvements may be challenging in Iran's context, smaller, manageable interventions and changing public perceptions could lead to meaningful progress. Policymakers should ensure that there are real consequences for risky driving and work towards fostering respect for law enforcement. This can be achieved by enforcing consistent penalties and increasing police presence in high-risk areas, which may help deter reckless driving behavior and improve overall compliance with traffic laws. Targeted enforcement with consistent law application and technology like traffic cameras can help reduce risky driving and combat the inconsistencies in law enforcement. To address personal traits, low-cost public education campaigns can raise awareness and promote safer driving behaviors.

**Limitations**

Since this study is qualitative in nature, it is crucial to acknowledge the inherent limitations associated with this approach. Despite implementing measures to enhance trustworthiness, it's important to recognize several limitations. Our findings are context-specific and may not apply to other populations or settings. The sample size was also relatively small, which may impact the representativeness of the findings. Additionally, participants' self-reporting of their experiences and perspectives introduces potential biases and subjectivity. Participants may downplay or exaggerate their risky driving to appear more socially acceptable, or they might forget incidents due to recall bias. This can lead to an inaccurate understanding of the true causes of risky driving. Thematic analysis gave us useful information about what makes people drive dangerously, but it's important to remember that this method may not show the whole picture because interpreting and putting together themes is subjective and affected by the researchers' views and biases. It is essential to view the findings of this study as a starting point for further exploration and understanding or added context to the preexisting knowledge.

The present study, which analyzed the status of pre-hospital emergency medication and equipment in Qazvin province, showed that the status of pre-hospital emergency medication and equipment is far from the standards. So only 56.09% of marks have been obtained. The highest score was in the medication and equipment section of the base, jam-bag equipment, and 

## Conclusion

In conclusion, this qualitative exploration shed light on the background and determinants of high-risk driving among passenger car and heavy vehicle drivers in Iran. By conducting interviews with high-risk drivers and analyzing their firsthand experiences and perspectives, this study found that economic challenges and job-related factors, such as low income, excessive work hours, time constraints, and poor working conditions, significantly contributed to RDB. Time pressure, especially among heavy vehicle drivers, is often imposed by employers, contract providers, or vehicle owners. Sociocultural factors, including attitudes toward law enforcement and peer interactions, also played a role. Personal traits such as anger, inexperience, and flawed risk perception were identified as influential factors. Additionally, this study emphasized the impact of law enforcement, road conditions, and environmental factors. The feeling of arbitrary and unfair law enforcement and environmental factors of hot weather and poor road conditions were relatively novel findings of this study that can be attributable to developing or underdeveloped settings with certain climates. 

These findings provide valuable insights for policymakers, traffic authorities, and driver training programs in developing targeted interventions to address the root causes of high-risk driving behavior and improve road safety in Iran. Future research employing quantitative methods and larger sample sizes to validate or modify our findings could provide a more comprehensive understanding of the determinants of high-risk driving behavior.

**Acknowledgement**

Financial support for this study was provided by Shiraz University of Medical Sciences under grant number 24853. The authors express their gratitude to all participants and those who played a role in facilitating the study.

**Authors Contributions: **RF, STH, and KBL contributed to the conception and design of the study. RF and SHA collected the data. SSh, AKS, and YS participated in the data analysis. RF, SHA, SSh and YS contributed to drafting the manuscript. KBL, STH, and AKS critically revised the manuscript for significant intellectual content. All authors approved the final version for publication and take responsibility for all aspects of the work.

## References

[1] Toroyan T (2009). Global status report on road safety. Inj Prev.

[2] Bank W. Road Deaths and Injuries Hold Back Economic Growth in Developing Countries. World Bank; Washington, DC; 2018.

[3] Sadeghian F, Mehri A, Ghodsi Z, Baigi V, Bardsiri M, Sharif-Alhoseini M (2023). Trends in road traffic injuries and mortality in the Islamic Republic of Iran in 1997–2020. East Mediterr Health J.

[4] Bucsuházy K, Matuchová E, Zůvala R, Moravcová P, Kostíková M, Mikulec R (2020). Human factors contributing to the road traffic accident occurrence. Transportation Research Procedia.

[5] Klauer SG, Guo F, Simons-Morton BG, Ouimet MC, Lee SE, Dingus TA (2014). Distracted driving and risk of road crashes among novice and experienced drivers. N Engl J Med.

[6] Jafarpour S, Rahimi-Movaghar V (2014). Determinants of risky driving behavior: a narrative review. Med J Islam Repub Iran.

[7] Batrakova A, Gredasova O (2016). Influence of road conditions on traffic safety. Procedia Engineering.

[8] Saha S, Schramm P, Nolan A, Hess J (2016). Adverse weather conditions and fatal motor vehicle crashes in the United States, 1994-2012. Environ Health.

[9] Mekker MM, Remias SM, McNamara ML, Bullock DM. Characterizing interstate crash rates based on traffic congestion using probe vehicle data. 2020; JTRP Affiliated Reports, Paper 31.

[10] Bandura A (1986). Social foundation of thought and action: A social cognitive theory. Prentice-Hall google schola.

[11] Azami-Aghdash S (2020). Meta-synthesis of qualitative evidence in road traffic injury prevention: a scoping review of qualitative studies (2000 to 2019).. Arch Public Health.

[12] Love S, Truelove V, Rowland B, Kannis-Dymand L, Davey J (2022). Is all high-risk behaviour premeditated? A qualitative exploratory approach to the self-regulation of habitual and risky driving behaviours. Transportation Research Part F: Traffic Psychology and Behaviour.

[13] Mazengia EM, Kassie A, Zewdie A, Demissie GD (2023). A qualitative study of perception related to risky driving behavior in Debre Markos City, North West Ethiopia, 2021. BMC Public Health.

[14] Watters SE, Beck KH (2016). A qualitative study of college students' perceptions of risky driving and social influences. Traffic Inj Prev.

[15] Day MR, Thompson AR, Poulter DR, Stride CB, Rowe R (2018). Why do drivers become safer over the first three months of driving? A longitudinal qualitative study. Accid Anal Prev.

[16] Shams M, Shojaeizadeh D, Majdzadeh R, Rashidian A, Montazeri A (2011). Taxi drivers' views on risky driving behavior in Tehran: a qualitative study using a social marketing approach. Accid Anal Prev.

[17] Graneheim UH, Lundman B (2004). Qualitative content analysis in nursing research: concepts, procedures and measures to achieve trustworthiness. Nurse Education Today.

[18] Cho J, Trent A (2006). Validity in qualitative research revisited. Qualitative Research.

[19] Johnson JL, Adkins D, Chauvin S (2020). A review of the quality indicators of rigor in qualitative research. Am J Pharm Educ.

[20] Guba EG, Lincoln YS (1994). Competing paradigms in qualitative research. Handbook of Qualitative Research.

[21] Atombo C, Wu C, Tettehfio EO, Agbo AA (2017). Personality, socioeconomic status, attitude, intention and risky driving behavior. Cogent Psychology.

[22] Mőller H, Rogers K, Cullen P, Senserrick T, Boufous S, Ivers R (2021). Socioeconomic status during youth and risk of car crash during adulthood. Findings from the DRIVE cohort study. J Epidemiol Community Health.

[23] Machado-León JL, de Oña J, de Oña R, Eboli L, Mazzulla G. Socio-economic and driving experience factors affecting drivers’ perceptions of traffic crash risk. Transportation Research Part F: Traffic Psychology and Behaviour. 2016;37:41-51.

[24] Rendon-Velez E, Van Leeuwen P, Happee R, Horvath I, van der Vegte WF, de Winter JC. The effects of time pressure on driver performance and physiological activity: A driving simulator study. Transportation Research Part F: Traffic Psychology and Behaviour. 2016;41:150-69.

[25] Cœugnet S, Naveteur J, Antoine P, Anceaux F. Time pressure and driving: Work, emotions and risks. Transportation Research Part F: Traffic Psychology and Behaviour. 2013;20:39-51.

[26] Lin CJ, Jia H (2023). Time Pressure Affects the Risk Preference and Outcome Evaluation. Int J Environ Res Public Health.

[27] Baumann M, Krems JF. Situation awareness and driving: A cognitive model. Modelling driver behaviour in automotive environments: Critical issues in driver interactions with intelligent transport systems. 2007: 253-65.

[28] Seward J, Stavrinos D, Moore D, Attridge N, Trost Z (2021). When driving hurts: characterizing the experience and impact of driving with back pain. Scand J Pain.

[29] Veldhuijzen DS, van Wijck AJ, Wille F, Verster JC, Kenemans JL, Kalkman CJ, et al. Effect of chronic nonmalignant pain on highway driving performance. Pain. 2006 May;122(1-2):28-35. 10.1016/j.pain.2005.12.019 16495013

[30] Dahlen ER, Martin RC, Ragan K, Kuhlman MM (2005). Driving anger, sensation seeking, impulsiveness, and boredom proneness in the prediction of unsafe driving. Accid Anal Prev.

[31] Heslop S, Harvey J, Thorpe N, Mulley C (2010). Factors that comprise driver boredom and their relationships to preferred driving speed and demographic variables. Transportation Planning and Technology.

[32] Greenfield TK, Rogers JD (1999). Alcoholic beverage choice, risk perception and self-reported drunk driving: effects of measurement on risk analysis. Addiction.

[33] Ulleberg P, Rundmo T (2003). Personality, attitudes and risk perception as predictors of risky driving behaviour among young drivers. Safety Science.

[34] Carter PM, Bingham CR, Zakrajsek JS, Shope JT, Sayer TB (2014). Social norms and risk perception: Predictors of distracted driving behavior among novice adolescent drivers. Journal of Adolescent Health.

[35] Ivers R, Senserrick T, Boufous S, Stevenson M, Chen HY, Woodward M, et al. Novice drivers' risky driving behavior, risk perception, and crash risk: findings from the DRIVE study. Am J Public Health. 2009 Sep;99(9):1638-1644. 10.2105/AJPH.2008.150367 PMC2724457 19608953

[36] Sayed I, Abdelgawad H, Said D (2022). Studying driving behavior and risk perception: a road safety perspective in Egypt. Journal of Engineering and Applied Science.

[37] Meng A, Siren A (2012). Cognitive problems, self-rated changes in driving skills, driving-related discomfort and self-regulation of driving in old drivers. Accid Anal Prev.

[38] Mohammadpour SI, Nassiri H (2021). Aggressive driving: Do driving overconfidence and aggressive thoughts behind the wheel, drive professionals off the road?. Transportation Research Part F: Traffic Psychology and Behaviour.

[39] Judge TA, Piccolo RF, Kosalka T (2009). The bright and dark sides of leader traits: A review and theoretical extension of the leader trait paradigm. The Leadership Quarterly.

[40] Scott-Parker B, Watson B, King MJ (2009). Understanding the psychosocial factors influencing the risky behaviour of young drivers. Transportation Research Part F: Traffic Psychology and Behaviour.

[41] Tontodonato P, Drinkard A (2020). Social learning and distracted driving among young adults. American Journal of Criminal Justice.

[42] Ouimet MC, Pradhan AK, Simons-Morton BG, Divekar G, Mehranian H, Fisher DL (2013). The effect of male teenage passengers on male teenage drivers: Findings from a driving simulator study. Accid Anal Prev.

[43] Shepherd JL, Lane DJ, Tapscott RL, Gentile DA (2011). Susceptible to Social Influence: Risky “Driving” in Response to Peer Pressure1. J Appl Soc Psychol.

[44] Oei TP, Kerschbaumer DM (1990). Peer attitudes, sex, and the effects of alcohol on simulated driving performance. Am J Drug Alcohol Abuse.

[45] Watters SE, Beck KH (2016). A qualitative study of college students' perceptions of risky driving and social influences. Traffic Inj Prev.

[46] Xu T, Zhu H, Xiong H, Zhong H, Chen E. Exploring the social learning of taxi drivers in latent vehicle-to-vehicle networks. IEEE Transactions on Mobile Computing. 2019 May 7;19(8):1804-17.

[47] Dussault C (1990). Effectiveness of a selective traffic enforcement program combined with incentives for seat belt use in Quebec. Health Education Research.

[48] Schafer JA, Mastrofski SD (2005). Police leniency in traffic enforcement encounters: Exploratory findings from observations and interviews. Journal of Criminal Justice.

[49] Naveen N, Yadav SM, Kumar AS (2018). A study on potholes and its effects on vehicular traffic. Int J Creat Res Thoughts.

[50] Lim J, Park S, Choi D, Bok K, Yoo J (2022). Road Speed Prediction Scheme by Analyzing Road Environment Data. Sensors (Basel)..

[51] Solmazer G, Üzümcüoğlu Y, Özkan T. The role of traffic law enforcements in the relationship between cultural variables and traffic fatality rates across some countries of the world. Transportation Research Part F: Traffic Psychology and Behaviour. 2016;38:137-50.

[52] Stanojević P, Jovanović D, Lajunen T (2013). Influence of traffic enforcement on the attitudes and behavior of drivers. Accid Anal Prev.

[53] Parker D, Manstead AS, Stradling SG, Reason JT, Baxter JS. Intention to commit driving violations: An application of the theory of planned behavior. Journal of Applied Psychology. 1992;77(1):94.

[54] Guggenheim N, Taubman-Ben-Ari O, Ben-Artzi E (2020). The contribution of driving with friends to young drivers' intention to take risks: An expansion of the theory of planned behavior. Accid Anal Prev.

[55] Chen CF, Chen CW (2011). Speeding for fun? Exploring the speeding behavior of riders of heavy motorcycles using the theory of planned behavior and psychological flow theory. Accid Anal Prev.

[56] Ram T, Chand K (2016). Effect of drivers’ risk perception and perception of driving tasks on road safety attitude. Transportation Research Part F: Traffic Psychology and Behaviour.

[57] Poulter DR, McKenna FP (2010). Evaluating the effectiveness of a road safety education intervention for pre-drivers: an application of the theory of planned behaviour. Br J Educ Psychol.

